# Latest Update on Outer Membrane Vesicles and Their Role in Horizontal Gene Transfer: A Mini-Review

**DOI:** 10.3390/membranes13110860

**Published:** 2023-10-26

**Authors:** Beatrice Marinacci, Paweł Krzyżek, Benedetta Pellegrini, Gabriele Turacchio, Rossella Grande

**Affiliations:** 1 Department of Pharmacy, University “G. d’Annunzio”, Chieti-Pescara, 66100 Chieti, Italy; beatrice.marinacci@unich.it (B.M.); benedetta.pellegrini@unich.it (B.P.); 2 Department of Innovative Technologies in Medicine & Dentistry, University of Chieti-Pescara, 66100 Chieti, Italy; 3 Department of Microbiology, Faculty of Medicine, Wroclaw Medical University, 50-368 Wroclaw, Poland; krojcerpawel@gmail.com; 4 Institute of Endocrinology and Experimental Oncology “Gaetano Salvatore” (IEOS), National Research Council, 80131 Naples, Italy; gabriele.turacchio@gmail.com; 5 Institute of Translational Pharmacology (IFT), National Research Council, 67100 L’Aquila, Italy; 6 Center for Advanced Studies and Technology (CAST), University “G. d’Annunzio”, Chieti-Pescara, 66100 Chieti, Italy

**Keywords:** outer membrane vesicles, extracellular vesicles, Gram-negative bacteria, horizontal gene transfer, vesiduction

## Abstract

Outer membrane vesicles (OMVs) are spherical, lipid-based nano-structures, which are released by Gram-negative bacteria in both in vitro and in vivo conditions. The size and composition of OMVs depend on not only the producer bacterial species but also cells belonging to the same strain. The mechanism of vesicles’ biogenesis has a key role in determining their cargo and the pattern of macromolecules exposed on their surface. Thus, the content of proteins, lipids, nucleic acids, and other biomolecules defines the properties of OMVs and their beneficial or harmful effects on human health. Many studies have provided evidence that OMVs can be involved in a plethora of biological processes, including cell-to-cell communication and bacteria–host interactions. Moreover, there is a growing body of literature supporting their role in horizontal gene transfer (HGT). During this process, OMVs can facilitate the spreading of genes involved in metabolic pathways, virulence, and antibiotic resistance, guaranteeing bacterial proliferation and survival. For this reason, a deeper understanding of this new mechanism of genetic transfer could improve the development of more efficient strategies to counteract infections sustained by Gram-negative bacteria. In line with this, the main aim of this mini-review is to summarize the latest evidence concerning the involvement of OMVs in HGT.

## 1. Introduction

Bacterial extracellular vesicles (EVs) are undoubtedly one of the most attractive topics, on which an increasing number of researchers are currently focusing their studies. Nowadays, it is widely known that both Gram-positive and Gram-negative bacteria can release these rounded, lipidic nanostructures in vitro as well as in vivo [1,2,3]. Their discovery dates back to 1965, after studying and observing the presence and release of vesicles from the outer membrane in *Escherichia coli* strains. Initially, it was thought that these structures were the result of a process of cell lysis, but over time, it became apparent that they blebbed from the outer membrane of bacterial surface without the need of lytic processes, giving rise to the so-called “outer membrane vesicles” (OMVs). Gamazo and Moriyon, in 1987, provided the first electron microscope images and analysis of their contents by SDS-PAGE focusing on the OMVs of *Brucella melitensis* [4]. For a long time, it was thought that only Gram-negative bacteria could produce extracellular vesicles, as Gram-positive bacteria are characterized by the presence of a thick cell wall and the absence of an outer membrane. Subsequently, studies conducted by Lee et al. (2009) on *Staphylococcus aureus* revealed, for the first time, that Gram-positive bacteria can also release vesicles into the extracellular environment with physical characteristics similar to those released by Gram-negative bacteria [5]. Classically, EVs can mediate cell-to-cell communication and deliver biomolecules to both other microorganisms and host cells [1]. At the same time, evidence suggests that EVs can have a broad variety of functions depending on the producing bacteria. Those released by probiotics, for example, exert a number of health-promoting effects, such as the regulation of intestinal homeostasis and immunomodulation, allowing the consideration of their use as health-promoting drugs [6]. On the other hand, EVs produced by pathogens are directly involved in enhancing bacterial survival and delivering virulence factors, thus exerting a harmful effect on the host, prompting great concern among scientists [7,8]. In addition to the intrinsic differences between the vesicles released by distinct bacterial species, EVs properties can be influenced by their biogenesis route, which determines their composition as well as cargo selectivity [9]. In this mini-review, we focus on the vesicles released by Gram-negative bacteria, namely, outer membrane vesicles (OMVs). Based on the literature published in the last six years concerning this topic, we discuss the characteristics and properties of OMVs highlighting their role in horizontal gene transfer (HGT).

## 2. Review Strategy

To search original papers to be analyzed and discussed in the current review, we used the PUBMED and Scopus databases. Only original articles from the last six years were included (1 January 2018–August 2023), and the keywords used in the bibliographic research were as follows: “OMVs”, “outer membrane vesicles”, “Gram-negative”, “horizontal gene transfer”, “biogenesis”, “composition”, “cargo”, and “characteristics”. As a result of these, 40 original articles are discussed in different sections of the review.

## 3. Biogenesis and Types of Extracellular Vesicles

It should be borne in mind that the production of EVs comes with a high price [10]. These structures are energy-intensive to produce as they are composed of complex macromolecules requiring a high metabolic input in their synthesis. Additionally, EVs released from microbial cells must overcome the stability of membranes of the bacterial producers. Despite this, the advantages resulting from the secretion of EVs outweigh the above disadvantages and, in the course of evolution, have contributed to the widespread dissemination and perpetuation of this phenomenon [10,11].

### 3.1. Factors Associated with Vesicles Formation

EVs have been studied for decades, and the ability to secrete them has been found in all species of microorganisms, but only recently have genetic and biochemical analyses allowed scientists to determine more precisely the mechanisms associated with their formation [7]. In the course of many years of research, it was found that the structure of fatty acids that build bacterial cell membranes seems to be of great importance in the biogenesis of EVs because these macromolecules affect the stiffness and fluidity of membranes and thus also the process of detachment of its fragments [10,12,13]. In this regard, the importance of polar lipid head groups, which determine the conformation of lipids, is also indicated. For example, it was shown that local or asymmetric accumulation/deficit of phosphatidylethanolamine in cell membranes dramatically affects their local parameters and may initiate or inhibit the vesiculation process [12,14,15]. Moreover, the presence of misfolded proteins, peptidoglycan fragments, or highly hydrophobic molecules also modulates this phenomenon [7,10,12,16]. The relationship between the membrane curvature and the vesiculation process is best described in *Pseudomonas aeruginosa* and their hydrophobic compounds involved in *quorum sensing*—*Pseudomonas* quinolone signals (2-heptyl-3-hydroxy-4-quinolone) [16,17,18]. In that case, the research results support the model of vesicle formation through an outer leaflet expansion, during which this amphiphilic molecule accumulates locally within the outer membrane of *P. aeruginosa*, extending the leaflet of this membrane and conditioning its invagination by destabilizing molecules of lipopolysaccharide (LPS), while having no effect on the inner membrane.

### 3.2. Mechanisms of Vesicles Biogenesis

**Figure 1:**
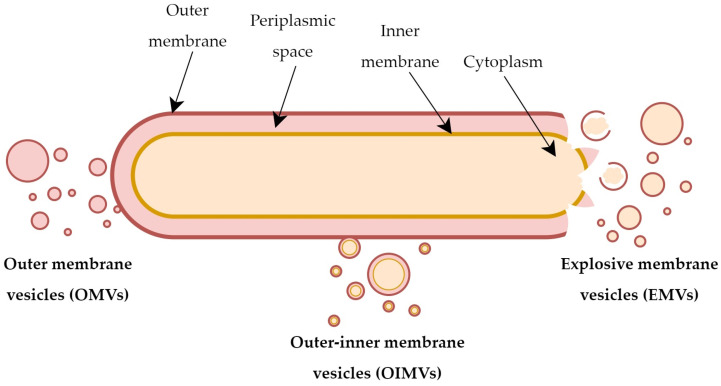
Types of extracellular vesicles produced by Gram-negative bacteria.

Many studies in recent years indicate that EVs differ in structure and chemical composition, even within bacteria of the same species/strains. It is postulated that different mechanisms of their development may be largely responsible for this diversity. Scientific evidence highlights the existence of two basic mechanisms being responsible for the formation of EVs in Gram-negative bacteria: (1) active secretion by living bacterial cells, resulting in the formation of OMVs, or (2) secretion stimulated by the activity of hydrolytic enzymes, giving rise to various subpopulations of vesicles, including outer–inner membrane vesicles (OIMVs) or explosive membrane vesicles (EMVs) [16] (Figure 1).

OMVs are an archetypal example of EVs of microbial origin [12,16]. OMVs are lipidic structures that detach from the outer membrane of Gram-negative bacteria and carry fragments of the periplasm and cell wall, as well as some of outer membrane components (lipids, proteins, and sometimes also nucleic acids) [12]. In most Gram-negative bacteria, the stability of cell envelopes is determined by various types of cross-linking interactions, including covalent bonds between peptidoglycan and Brown’s lipoprotein (Lpp) or non-covalent bonds between peptidoglycan and the outer membrane protein OmpA [19,20]. For this reason, the total number of this type of bonds often correlates inversely with the vesicles’ secretion capacity [7]. On the other hand, in some bacteria, it is observed that the increased ability to produce OMVs is a consequence of a generalized stress response to high concentrations of toxic metabolites or abnormally formed proteins. After their local accumulation within the cell membrane, it undergoes local invagination and leads to the extracellular release of OMVs with toxic byproducts [21].

Gram-negative bacteria are also capable of producing another subpopulation of EVs called OIMVs [10,16]. Unlike OMVs, these structures consist of two layers—the inner and the outer membrane. For this reason, OIMVs contain not only components typical for OMVs but also components of the inner membrane, such as ATP molecules, nucleic acid strands, and other cytoplasmic macromolecules [10]. The formation of OIMVs most likely occurs as a result of local weakening of the murein structure by hydrolytic enzymes (e.g., autolysins), conditioning the insertion of the inner membrane into the periplasm. This mechanism allows the cytoplasm carrying nucleic acids or ribosomal subunits to reach the interior part of the forming vesicle and detachment from the outer membrane, taking its fragment with it [10,16]. Many experimental studies have shown that numerous species of Gram-negative bacteria are capable of producing OIMVs and that nucleic acid particles are specifically transported by this subpopulation of vesicles [16].

Explosive cell lysis is another, alternative route of secretion of EVs [22]. This process most often occurs as a result of rapid destruction of the genetic material of the microorganism. The consequence of this phenomenon is the destabilization and disruption of the cell membrane, which further spontaneously recirculates creating explosive membrane vesicles (EMVs) [12,16]. Studies supporting this model of vesicles’ development include results showing stimulation of the EMVs’ secretion during, e.g., a prophage-dependent activation of endolysins in *Escherichia coli* [23] or *Shewanella vesiculosa* [24]. However, little is known about the frequency and biological importance of this process among Gram-negative bacteria.

## 4. Components of OMVs and Their Biological Functions

In Gram-negative bacteria, OMVs are important as they play an essential role in several biological processes, including virulence, HGT, export of cellular metabolites, contracting of phage infections, cell-to-cell communication, and modulation of the host immune response. OMV production occurs at the constitutive level for a wide variety of Gram-negative bacteria, suggesting that it is a highly conserved process, and furthermore, their production has been found in a variety of environments, including both planktonic cultures and biofilms. In addition, the levels and quantities of the vesicles can be altered by many factors, such as the temperature, nutrient availability, oxidation, cell density, and presence of envelope-targeting antibiotics [25,26,27,28]. In general terms, OMVs are surrounded by a single phospholipidic bilayer derived from the outer membrane and are primarily composed of LPSs, membrane phospholipids, and outer membrane proteins (OMPs), while inside, they contain different biomacromolecules [9,16,29]. Scientists are currently linking the OMVs’ cargo with physiological functions performed by these structures; therefore, a more in-depth look at their biochemical profile may provide substantial information about their biological role.

The main interest in pathogenic OMVs has been driven by the transport of numerous cargo molecules, including virulence factors, adhesins, toxins, DNA, RNA, and immunomodulatory factors. Examples are the OmpA family proteins (OrpF) of *Pseudomonas aeruginosa*, the vacuolating cytotoxin (VacA), and the porin HopA in *Helicobacter pylori*. The interaction of the bacteria with the host triggers the release of OMVs containing various proteins, particularly adhesion molecules, as was well proven for *H. pylori* OMVs delivering the sialic acid–binding adhesin (SabA) and blood group antigen-binding adhesion (BabA) [30,31], but also toxins and other virulence factors helping the bacteria to invade host cells and evade the host’s defense system, modulating its immune response (Table 1) [32,33,34]. The modes of entry of OMVs into host cells are diverse but mostly occur by endocytosis mediated by clathrin, caveolin, and lipid rafts and tend to be influenced by the length of the LPS and the composition of the cell membrane [35,36].

As previously mentioned, OMVs’ functions are far from being limited to pathogenicity, and the role of these carrier structures has proven to be more complex than initially expected. In particular, differences in the peptidoglycan composition cause some bacteria to be more prone to death than others, such as *Lysobacter* spp., which, by producing endopeptidases (L1, L4, and L5), amidase (L2), and muramidase (L3), are able to degrade competing Gram-negative bacteria [37]. Furthermore, OMV-associated toxins are more potent than their soluble form as they are protected from cellular proteases [38]. Among the more versatile functions, some enzymes packaged within OMVs play an important role. For example, Ronci et al. (2019) showed that the α-carbonic anhydrase (αCA), which is important for *H. pylori* survival in the human stomach, can be detected in its OMVs, in both planktonic and biofilm phenotypes, and the authors speculated that the presence of this enzyme could be correlated with the release of extracellular DNA (eDNA) [39]. Other authors have shown that *Pseudomonas putida*, a bacterium that colonizes the soil, in the presence of lignin produces many extracellular vesicles containing various enzymes, especially those involved in the β-ketoadipate pathway, which is important in the catabolism of aromatic compounds, and have proposed a possible mechanism of nutrient acquisition and/or OMV-mediated catabolism of toxic substrates [40].

### 4.1. OMV-Associated DNA

The presence of DNA in OMVs can be traced back to about 1989, which is when DW Dorward and his colleagues began discussing the DNA present in *Neisseria gonorrhoea* vesicles [41]. Since then, an increasing number of studies have become available in the literature describing the presence and function of chromosomal and/or plasmid DNA in the extracellular vesicles of various pathogens. The packaging of DNA into bacterial vesicles could occur as a spontaneous consequence of cell lysis. As hypothesized by Turnbull and colleagues, after the release of the cytoplasmic content into the extracellular space, all the components are randomly encased within the vesicles formed after the merging of membrane fragments [22]. On the contrary, Bitto et al. suggested that bacterial cells, when encountering cell division, can store DNA inside vesicles as the result of an active and regulated mechanism [42]. The functions of DNA delivered by bacterial vesicles are diverse, but the most analyzed aspects are both the transfer of genes for antibiotic resistance, such as the carbapenemase OXA-24 in *Acinetobacter baumannii* to confer resistance to carbapenems, and the exchange of genetic material between and within species, as seen in *Escherichia coli* O157:H7 [43,44]. In addition to the above, eDNA has been shown to be present not only inside OMVs but also on their surface. Grande et al. in 2015, using transmission electron microscopy (TEM) and DNase I-gold marking, demonstrated the presence of eDNA associated with vesicles produced by *Helicobacter pylori* in both planktonic and biofilm phenotypes. In particular, the authors demonstrated that it is involved in biofilm formation [45]. Subsequently, in 2017, Bitto et al. demonstrated the abundant DNA presence on the surface of other pathogens such as *Salmonella typhimurium*, *Porphyromonas gingivalis*, and *Pseudomonas aeruginosa* [42]. However, the studies of the last six years regarding the role of vesicle-borne DNA will be described in the dedicated section.

### 4.2. Potential Applications of OMVs

In recent years, OMVs have been studied for their potential use in the biotechnology industry. In particular, OMVs are good potential candidates for vaccines and drug delivery as they are easily absorbed by cells and could have immunomodulatory effects. Indeed, in 2019, Scaria et al. investigated OMVs produced by *Neisseria meningitidis* as a vehicle for a protein that can induce antibody production to prevent malaria transmission, while a recent study in mouse models by Zare Banadkoki and colleagues showed that *Pseudomonas aeruginosa* PAO1 vesicles, conjugated with diphtheria toxoid (DT), significantly increased IgG levels in immunized mice and thus could be used for vaccine development. The latter framework also includes studies such as the recent one by Weyant et al., in which methods for the engineering of OMVs are proposed [46,47,48,49].

**Table 1:** Virulence factors and activities associated with OMVs released by Gram-negative bacteria.

Bacterial Species	Vesicle-Associated Virulence Factors	Activity	Cytotoxic Activity	Reference
*Pseudomonas aeuriginosa* (clinical strains DH1137)	Orp F, OrpH	Modulation of the host innate immune response: ↓ expression of several genes belonging to the Major Histocompatibility Complexes (MHC) class II ↓ proteins important for antigen presentation to T-helper lymphocytes, such as CD74	*ND*	[30]
*Helicobacter pylori* (NCTC11637; Hp-400)	CagA, VacA, UreB	↑ release of inflammatory factors related to bacterial infection including IL-6, IFN-γ, IL-8, and TNF-α	*ND*	[50]
*Actinobacillus pleuropneumoniae* (MIDG2331 and mutants)	ApxIII toxin, MomP2, OmlA, FkpA, OmpP1, LpoA	Immunomodulatory effects evaluated in vitro: ↓ non-specific immune response by inhibiting the expression of several genes normally overexpressed during the innate immune response (e.g., chemokines and IL-6)	*ND*	[51]
*Bordetella pertussis* (Tohama I strain CIP 81.32 and isogenic mutant BpΔCyaA)	Adenylate cyclase toxin (CyaA), pertussis toxin (Ptx), SodB, KatA, AhpC, AhpD	Direct interaction with macrophages: ↓ expression of genes important in the macrophage response to bacterial infection, which leads to the persistence of the producing bacterium within macrophages and thus increased survival of the pathogen itself	*ND*	[52]
*Borrelia burgdorferi* B31 (ATCC, 35210, and GCB726)	Outer membrane protein (OspA, OspB, OspC)	OMVs represent a vehicle to evade the immune system and could explain the persistence of the infection.	No cytotoxicity reported against non-immune cells (skin fibroblasts and chondrosarcoma cells)	[53]
*Vibrio cholerae* (WT and mutant strains)	CT toxin, OmpU, OmpT	OMVs act as a protective envelope for cholera toxin (CT), which, when internalized in the intestinal cells, undergoes degradation by intestinal proteases.	*ND*	[38]
*Burkholderia cepacia* (ATCC 25416)	OmpW, OmpA, Type 1 fimbrial protein, A chain, TPR repeat family protein, lipase, protease	Pro-inflammatory action at small doses in vitro: ↑ expression of genes coding for pro-inflammatory cytokines	Cytotoxic effects on human lung cells A549	[54]
*Escherichia coli* O78:H11 (ATCC 35401)	Colonization factor I (CFA/I), heat-labile enterotoxin (LT), and non-classical factors: EtpA, EatA, and TibA	Stimulation of immune responses: ↑ release of neutralizing antibodies stimulated by the LT B immunogenic subunit expressed on OMVs. Stimulation of a Th1 immune response in macrophages: ↑ expression of CD40, MHCII, CD80, CD86; ↑ release of IL-6 and MCP-1	No cytotoxicity detected in RAW 264.7 cells for 48 h	[55]
*Treponema denticola*	Msp	Inhibition of neutrophils chemotaxis; ↓ pPTEN levels; ↑ phosphatase activity of PTEN; ↓ PIP_3_ levels	*ND*	[56]

Abbreviations: *ND*, no data; ↑, increase; ↓, decrease.

## 5. Characterization of OMVs

**Figure 2:**
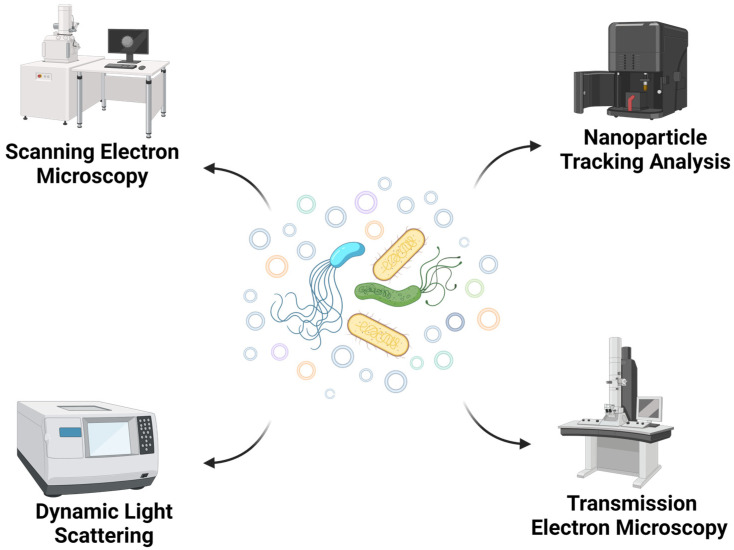
The most frequently applied methods for OMV characterization (created with Biorender.com, accessed on 19 September 2023).

**Figure 3:**
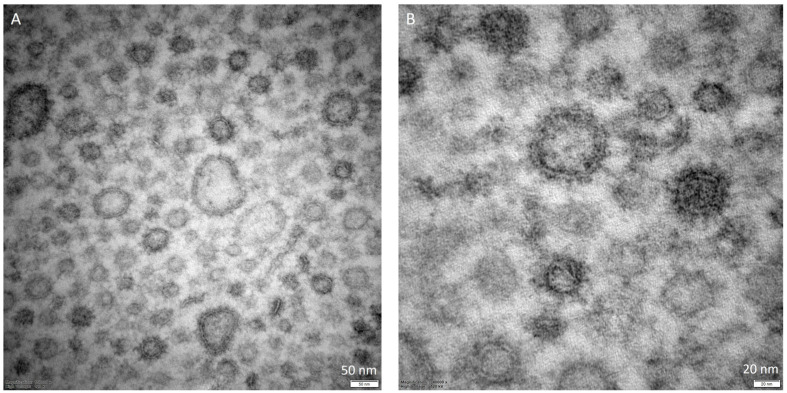
TEM representative images of *Porphyromonas gingivalis* extracellular vesicles isolated from the planktonic phenotype at 6 days of incubation. EPON embedded samples. Section thickness: 100 nm. (**A**) magnification: 150,000×; (**B**) magnification = 340,000×.

While describing the OMVs, another interesting aspect to examine is their morphological characterization. Considering the studies that constitute the core of this review, we reported in Table 2 the collection of data related to the main characteristics mentioned by the authors. The most frequently applied methods are nanoparticle tracking analysis (NTA), dynamic light scattering (DLS), and transmission electron microscopy (TEM) (Figure 2). Using an NTA protocol, it is possible to determine the size distribution and the concentration of the vesicles with high resolving capabilities [57,58], while DLS is suitable for measuring multiple physical attributes of particles; thus, they are often considered two complementary and orthogonal methods [59,60]. On the other hand, TEM is useful for its capability to detect and characterize single vesicles, despite the required protocol possibly affecting the size and morphology of the sample (Figure 3) [61]. In relation to the data reported in Table 2, it can be observed that in numerous works, the authors decided to combine different techniques in order to obtain more accurate results.

Looking closer to the diameter dimensions listed in the Table 2, it is noticeable that the size of the OMVs is quite variable. This difference could be occasionally related to the different techniques used for the characterization but can also be strictly associated to interspecific as well as intraspecific variety. It is worth noting that OMVs’ sizes and amounts can be influenced by specific growing conditions. For example, Bielaszewska and colleagues demonstrated that under simulated ileal environment medium (SIEM) and simulated colonic environment medium (SCEM), *Escherichia coli* O104:H4 strain C227-11ϕcu heightened the production of OMVs corresponding to 18.5- and 16.6-fold increases in particles/mL compared with the control in LB medium [62]. Similar outcomes were observed for the avian pathogenic *Escherichia coli* SCAO22, which, under the stimulus of amoxicillin and enrofloxacin treatment, significantly increased the amount of vesicles while reducing their dimensions [63]. At the same time, Kim et al. (2020) characterized via NTA the number and size of OMVs produced by *Burkholderia cepacia* ATCC 25416 when cultured under subinhibitory concentrations of antibiotics, namely, ceftazidime (CAZ), meropenem (MEM), and trimethoprim-sulfamethoxazole (SXT). As reported in the study, the size of the OMVs of *Burkholderia cepacia* appeared to be quite similar in the presence and absence of antibiotics, but in contrast, the antibiotics led to an increase in OMV production [64].

**Table 2:** Physical characteristics of OMVs.

Bacterial Producer	Technique	Diameter	Amount	Observations	Reference
*Escherichia coli* O104:H4 strain C227-11ϕcu	NTA	~100–130 nm	1 × 10^11^–1.5 × 10^12^ particles/mL (different growing conditions)	-	[62]
*Klebsiella pneumoniae* R1	EM	40–60 nm	-	Spherical morphology	[65]
*Klebsiella pneumoniae*-pGR and *Klebsiella pneumoniae*-PRM	TEM; DLS	113.8 ± 53.7 nm and 94.13 ± 41.10 nm	-	Uniform spherical morphology	[66]
Hypervirulent *Klebsiella pneumoniae* (hvKp) and ESBL-producing classical *K. pneumoniae* (cKp)	TEM; NTA	54–634 nm (median size 112 nm) and 17–523 nm (median size 78 nm)	~6.5 × 10^7^ particles/mL, ~3.5 × 10^7^ particles/ml	Oval and spherical morphologies	[67]
*Helicobacter pylori* NCTC11637 and Hp-400	TEM; NTA	50–250 nm	-	Spherical bilayerd morphology and cup-shaped structure	[50]
Carbapenem-resistant and hypervirulent *Klebsiella pneumoniae* NUHL30457	DLS; TEM	50–250 nm (median size of 132 nm)	-	Spherical bilayered structures	[68]
*Bordetella pertussis* BpAR106	TEM	50–25 nm	-	-	[28]
Avian pathogenic *Escherichia coli* SCAO22	TEM; nFCM	79.42 nm (control); 0.14 nm and 64.18 nm (under antibiotic treatment)	2.26 ± 0.78 × 10^10^ particles/mL (control)–5.66 ± 1.2 × 10^12^ particles/mL and 8.89 ± 0.36 × 10^11^ particles/mL (under antibiotic treatment)	Classic saucer-like vesicles	[63]
Carbapenem-resistant *Klebsiella pneumoniae*	TEM; DLS	68.1 to 396 nm (control); 78.8 to 396 nm (under antibiotic treatment)	-	Spherical morphology	[69]
*Escherichia coli* ATCC8739	DLS; TEM	48 ± 3 nm (at 37 °C); 37 ± 4 nm (at 27 °C); 24 ± 2 nm (at 20 °C)	-	Spherical morphology	[25]
*Klebsiella pneumoniae* hvK2115 and CRK3022	NTA; TEM	50–200 nm	9.1 × 10^11^ particles/mL and 2.6 × 10^11^ particles/mL	Spherical morphology	[70]
*Avibacterium paragallinarum* P4chr1	TEM	30–100 nm	-	Spherical morphology	[71]
*Pseudomonas aeruginosa* PAO1; PAO1 Δlys and PAO1 Δlys pJN105 lys	TEM; NTA	50–400 nm	Lower amount produced by bubbling compared with explosive cell lysis	Spherical morphology	[34]
*Bordetella pertussis* Tohama I strain CIP 81.32 (Bp) and BpΔCyaA (ΔCyaA)	TEM	10–240 nm (median size of 92.8 nm)	-	Spherical morphology with a uniform size distribution	[52]
*Pseudomonas aeruginosa* PAO1 and PW2884	NTA;TEM	178 nm, median size 119 nm (WT); 144 nm, median size 160 nm (PW2884)	1.29 × 10^9^ particles/mL (WT); 0.58 × 10^9^ particles/mL (PW2884)	-	[33]
*Helicobacter pylori* 26695 (ATCC 700392)	SEM	10–300 nm	-	Spherical morphology	[32]
*Burkholderia cepacia* ATCC 25416	NTA	129.7 ± 0.8 nm (control); under subinhibitory concentrations of antibiotics: MEM = 127.6 ± 1.2 nm; CAZ = 123.4 ± 2.5 nm; SXT = 154.9 ± 7.2 nm	2.79 × 10^9^ particles/mL (control); 2.45 × 10^10^ particles/mL (MEM); 1.91 × 10^10^ particles/mL (CAZ); 3.58 × 10^9^ particles/mL (SXT)	-	[64]
*Bordetella pertussis* B213 and *Bordetella bronchiseptica* BB-D09-SR	TEM	10–80 nm (after heat shock)	-	-	[26]
*Borrelia burgdorferi* B31 (ATCC, 35210) and GCB726	TEM	Four size categories: 0–20, 20.1–60, 60.1–100, and 100.1–140 nm	-	-	[53]
*Escherichia coli* O78:H11 (ATCC 35401)	PCS	50–300 nm	-	-	[55]
*Pseudomonas aeruginosa* DH1137	TEM; NTA	30–600 nm	-	Concave aspect	[30]
*Actinobacillus pleuropneumoniae* WT and mutant strains	Cryo-TEM	20–200 nm	-	Some WTs OMVs show a stick shape; OMVs of irregular shape in mutants	[51]
*Pseudomonas aeruginosa* PAO9503 and PAO9505	NTA; TEM	50–500 nm; in larger quantities 100–200 nm	-	-	[27]

Abbreviations: NTA, nanoparticle tracking analysis; DLS, dynamic light scattering; TEM, transmission electron microscopy (TEM); EM, electron microscopy; nFCM, flow nanoanalyzer; SEM, scanning electron microscopy; PCS, photon correlation spectroscopy; Cryo-TEM, cryo-transmission electron microscopy.

## 6. OMV-Mediated Horizontal Gene Transfer

**Figure 4:**
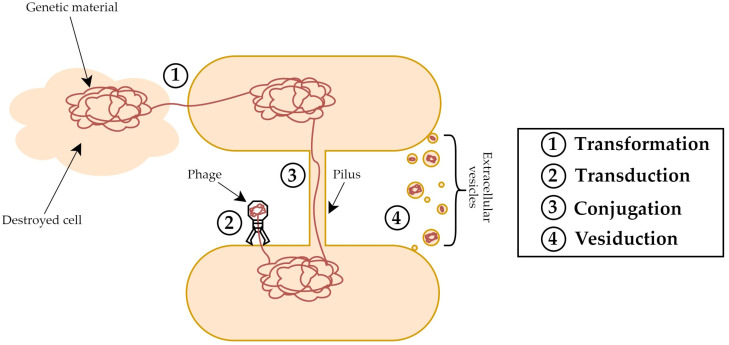
Four mechanisms involved in horizontal gene transfer (HGT).

Horizontal gene transfer (HGT) is a common process strictly related to the evolution of prokaryotes, which allows the exchange of genetic material between bacteria belonging even to different species. Normally, the sharing of genes leads to the development of a beneficial phenotype and thus supports bacterial survival [72]. To date, there are three mechanisms of HGT that have been extensively described. The first one is transformation, which involves an uptake of free DNA from the environment; the second is called conjugation and requires a physical connection between microbial cells; and the third is transduction and depends upon phages that deliver the genetic material during the infection of bacterial cells [72,73] (Figure 4). Nevertheless, the growing body of literature concerning bacterial extracellular vesicles suggests that they could constitute a new mechanism of HGT. One of the pioneering works about this topic was published in 1983 by Kahn and colleagues who identified “specialized membranous extensions termed transformasomes” that “are formed de novo during competence development and are responsible for selective uptake and protection of transforming DNA”. In this study, the transformation of *Haemophilus influenzae* was investigated, and the authors reported that donor DNA, packaged within the transformasomes, was protected from restriction and cellular degradative enzymes, thus facilitating the transformation process [74]. Similarly, in early 1989, the release of membrane vesicles, termed “blebs”, by *Neisseria gonorrhoeae* was investigated, and the authors reported that chromosomal DNA, plasmids, and RNA could be detected within these structures, which could be involved in the genetic exchange among bacteria [41]. Recently, in their study, Soler et al. proposed the term “vesiduction” to define the DNA transfer mediated by extracellular vesicles and introduce a new concept for the description of this non-canonical way of HGT [73] (Figure 4). The latest evidence from the last six years regarding the role of OMVs in HGT is presented in Table 3.

In 2021, Dell’Annunziata and colleagues were among the first to describe the transfer of plasmids via *Klebsiella pneumoniae*-derived OMVs. In this study, *K. pneumoniae* ATCC 10031 was transformed to obtain two distinct donor strains, namely, *K. pneumoniae*-pGR and *K. pneumoniae*-PRM, which contained a high-copy-number plasmid and a lower-copy-number plasmid, respectively. Both plasmids harbored genes that conferred resistance to ampicillin. In order to evaluate the OMV-mediated gene transfer, the recipient strains were incubated in the presence of purified vesicles, and the new bacterial colonies were screened using PCR analysis. All the recipient strains acquired β-lactam resistance. The findings of the research team allowed them to conclude that interspecies gene exchange via OMVs may occur, and that its efficiency is directly related to the plasmid copy number [66].

Similarly, Qiao et al. demonstrated that OMVs released by *Escherichia coli* DH5α delivered the plasmid pET28a-*nirS*—encoding for a nitrite reductase—which was successfully transferred and expressed into *E. coli* BL21. The transformation experiments, performed in the presence of different amounts of OMVs, indicated that the transformation frequency reached the highest value when the OMV dosage was the maximum tested. Furthermore, by staining the OMVs’ membranes and bacterial cell membranes with different fluorescent dyes, the authors were able to assert that vesicles were directly absorbed on the surface of *E. coli* BL21 [75].

Investigations performed some years age have described the effect of glycine on the bacterial membrane integrity, leading to the conclusion that this amino acid is capable of inhibiting peptidoglycan (PG) synthesis [76,77]. Based on these findings, other interesting results were obtained in a study by Aktar et al., wherein the factors that influence the incorporation of the DNA in the vesicles were investigated, and the knowledge of glycine’s role was deepened. The analysis of the OMVs released by *E. coli* BW25113 wild type (WT) and by three high OMV-producing strains (∆*nlpI*, ∆*rseA*, ∆*tolA*) revealed that alterations of PG increased the loading of DNA into OMVs, and glycine can be considered a stimulator of OMVs production as well as an enhancer of eDNA release. Moreover, the production of OIMVs from *E. coli* ∆*nlpI* and WT was also reported. Despite the lack of experiments focused strictly on the transfer of genetic material to recipient bacteria, this study provides new insights into the mechanisms of vesicle-mediated HGT [78].

Another important aspect to consider when discussing HGT is the environmental conditions that might influence the OMV-mediated gene transfer. Bielaszewska et al. proved that *E. coli* O104:H4 strain C227-11ϕcu could release OMVs containing the pESBL plasmid and successfully transfer them to different Enterobacteriaceae isolates, such as *Escherichia coli*, *Salmonella enterica*, *Klebsiella pneumoniae*, etc. The pESBL carried *bla*_CTX-M-15_ and *bla*_TEM-1_ genes, which conferred antibiotic resistance—ESBL phenotype—to the receiving cells. To better characterize this process, they performed the OMV isolation under simulated intraintestinal conditions and under antibiotic stress, comparing them with standard laboratory conditions. From their data, it can be assumed that the intraintestinal environment might increase the OMV-associated DNA content and the frequency of *bla*_CTX-M-15_ transfer. In addition, the frequency of gene transfer was also heightened up to 100 times, when OMVs produced in the presence of ciprofloxacin were tested. An explanation of this outcome could be the activation of the SOS response, which is a consequence of the antibiotic-mediated DNA damage, followed by increased OMVs production [62]. Likewise, Li et al. found that the transfer frequency of OMVs produced by *E. coli* SCAO22 was directly related to antibiotic exposure, especially enrofloxacin. Furthermore, antibiotic treatment could also correlate with morphological changes in OMVs, including diameter, nucleic acid, and protein concentration [63]. Despite the need for further research on the topic, these findings suggest that the effect of antibiotics on OMVs production could lead to the revelation of new pathways of antibiotic resistance spreading via HGT [62,63].

It is worth recalling, however, that not every OMV-mediated transfer is successful in inducing a resistant phenotype. For example, Xu and colleagues conducted a series of experiments evaluating the capacity of *Avibacterium paragallinarum* OMVs to transmit antibiotic resistance genes (ARGs) to a sensitive strain, namely, *A. paragallinarum* Modesto. The MIC of the transformed cells did not increase compared with the susceptible strain Modesto, although the PCR results confirmed that those colonies had amplification match ARG products. As assumed by the authors, this phenomenon was associated with a low gene recombination, where the ARGs were not integrated in the chromosomes, leading to a limited persistency of the transformation in the receiving strain [71]. These results differ from those illustrated by Chen et al., who revealed that the OMVs of a carbapenemase-2-producing *K. pneumoniae* (KP-R1) might induce a stable resistance in an antibiotic-susceptible strain of *K. pneumoniae*, and this acquired property was maintained after the third generation [65]. The contrasting observations among these studies highlight that there is a growing need for supplementary investigations so as to identify the variables that can impact the transfer process. In this context, the work of Tang et al. reveals additional elements by which OMV-mediated HGT mechanisms can be characterized. After proving that carbapenem-resistant *K. pneumoniae* can induce the spread of resistance genes by releasing OMVs, they observed that the gene transfer frequency varied among the recipient bacteria, suggesting a possible strain-dependent correlation [69].

Last, of particular interest are the conclusions of Wang and co-workers, who evaluated the ability of recipient cells of *K. pneumoniae* and *E. coli* to acquire both virulence and resistance plasmids delivered by *K. pneumoniae* OMVs. By screening the different products after the genetic transfer, information that OMVs could promote the intraspecies as well as the interspecies HGT was also demonstrated [70].

Considering all the studies cited above, it can be concluded that OMV-mediated HGT depends on numerous variables; thus, its characterization is a quite challenging issue. For example, in addition to the intrinsic differences between bacterial strains and species, several authors reported that the loading of DNA into vesicles could be influenced by the plasmid type and size along with protein binding and location within the cells [79]. Additionally, as reported by Li et al., another key point could be the expression of putative effectors that recruit OMVs on recipient cells’ surfaces [80]. Unquestionably, OMV-mediated HGT is a useful process conferring advantages to bacterial populations, and taken together, these findings provide new suggestions for further exploration of this topic, which are required to understand its role in health and disease.

**Table 3:** Implication of OMVs in Horizontal Gene Transfer.

Bacterial Producer	Genetic Material	Recipient Bacteria	Observations	References
*Avibacterium paragallinarum* P4chr1	ARGs: bl2d_oxa1; aph33ib; cml_e3; tetB	*A. paragallinarum* Modesto	- Antibiotic-sensitive *A. paragallinarum* Modesto survived on antibiotic-treated agar plates. - The MIC values in the transformed strains did not increase compared with the Modesto strain, suggesting that the genes transferred by OMVs are not persistent in the recipient cells.	[71]
*Escherichia coli* O104:H4 strain C227-11ϕcu	pESBL plasmid: blaCTX-M-15 and blaTEM-1	Clinical Enterobacteriaceae isolates and *E. coli* K-12 C600	- ↑ OMV-associated DNA content and ↑ frequency of OMV-mediated blaCTX-M-15 transfer under simulated intraintestinal conditions compared with laboratory conditions - ↑ frequency of OMV-mediated blaCTX-M-15 transfer in the presence of ciprofloxacin compared with control samples	[62]
*Escherichia coli* strains	pUC19; pCP20	-	- Peptidoglycan defects increase the incorporation of plasmids into OMVs. - Glycine is a stimulator of OMV production and the incorporation of plasmids into OMVs.	[78]
*Klebsiella pneumoniae* R1	blaKPC-2	*K. pneumoniae* S1; *E. coli* S1	- OMVs derived from a carbapenem-resistant strain of *K. pneumoniae* might induce resistance in the carbapenem-sensitive *K. pneumoniae* strain. - No evidence of the emergence of resistant *E. coli* species after OMV exposure	[65]
Hypervirulent *Klebsiella pneumoniae* (hvKp)	pLVPK-like plasmid: prmpA and iroB	ESBL-producing classical *K. pneumoniae* (cKp)	- Transformants of *K. pneumoniae* showed hypervirulent and multi-drug-resistant phenotypes.	[67]
*Klebsiella pneumoniae*-pGR and *Klebsiella pneumoniae*-PRM	Plasmids containing genes for β-lactamase: pGR and PRM	*K. pneumoniae* ATCC 10031; *E. coli* ATCC 25922; *S. enterica* ATCC 14028; *P. aeruginosa* ATCC 13388; and *B. cepacia* ATCC 25416	- Recipient cells of *K. pneumoniae* and other bacterial genera acquired resistance to ampicillin - The transformation efficiency is strictly dependent on the plasmid copy number.	[66]
Carbapenem-resistant and hypervirulent *Klebsiella pneumoniae* NUHL30457	Plasmids containing virulence and antimicrobial resistance genes	*K. pneumoniae* ATCC 700603	- Recipient cells of *K. pneumoniae* ATCC strain acquired resistance to carbapenem and potassium tellurite; the transformants had drug resistance and highly pathogenic phenotypes.	[68]
Avian pathogenic *Escherichia coli* SCAO22	IncI2 plasmid: blaCTX-M-55	*E. coli* C600	- OMVs mediated the transfer of the plasmid to the recipient bacterium. - The transfer frequency could be influenced by antibiotic treatment.	[63]
*Escherichia coli* DH5α	pET28a plasmid: nirS	*E. coli* BL21	- The cell-free OMVs were directly absorbed on the cell membrane of *E. coli* BL21. - The aerobic denitrification gene nirS can be transferred and expressed into the recipient bacterium. - ↑ OMVs dose leads to ↑ frequency of HGT.	[75]
Carbapenem-resistant *Klebsiella pneumoniae*	IncFIBpKPHS1 plasmid: blaNDM-1	*K. pneumoniae* ATCC 10031, ESBL-producing *K. pneumoniae* ATCC 700603, and hypervirulent *K. pneumoniae* NTUH-K2044	- OMV-mediated HGT could be considered a mechanism related to the emergence of CR-hvKP. - The gene transfer frequency could be strain-dependent.	[69]
*Klebsiella pneumoniae* hvK2115 and CRK3022	phvK2115 plasmid: rmpAp, rmpA2p and iroB; pCRK3022 plasmid	*E. coli* EC600 and *K. pneumoniae* K20809	- OMVs could mediate the intraspecific and interspecific HGT of the two tested plasmids.	[70]

Abbreviations: ARGs, antibiotic resistance genes; MIC, minimum inhibitory concentration; ESBL, extended spectrum beta-lactamases; HGT, horizontal gene transfer; CR-hvKP, carbapenem-resistant hypervirulent *Klebsiella pneumoniae*; ↑, increase; ↓, decrease.

## 7. Conclusions

Outer membrane vesicles released by Gram-negative bacteria are, without any doubts, currently among the most interesting topics in the microbiological field. With a growing body of experimental studies, numerous authors have contributed to the building of a solid description of the OMVs’ biogenetic processes, allowing for a better understanding of their involvement in multiple health and disease-associated conditions. The results presented in this mini-review highlight emerging novelties regarding the OMV-mediated HGT, although their limited amounts indicate also a clear need to increase interest in this topic.
